# Multiscale Modeling Indicates That Temperature Dependent [Ca^2+^]_i_ Spiking in Astrocytes Is Quantitatively Consistent with Modulated SERCA Activity

**DOI:** 10.1155/2015/683490

**Published:** 2015-08-04

**Authors:** Niko Komin, Mahsa Moein, Mark H. Ellisman, Alexander Skupin

**Affiliations:** ^1^Luxembourg Centre for Systems Biomedicine, University of Luxembourg, 7 Avenue des Hauts-Fourneaux, 4362 Esch-sur-Alzette, Luxembourg; ^2^National Centre for Microscopy and Imaging Research, University of California San Diego, 9500 Gilman Drive, La Jolla, CA 92093-0608, USA

## Abstract

Changes in the cytosolic Ca^2+^ concentration ([Ca^2+^]_i_) are the most predominant active signaling mechanism in astrocytes that can modulate neuronal activity and is assumed to influence neuronal plasticity. Although Ca^2+^ signaling in astrocytes has been intensively studied in the past, our understanding of the signaling mechanism and its impact on tissue level is still incomplete. Here we revisit our previously published data on the strong temperature dependence of Ca^2+^ signals in both cultured primary astrocytes and astrocytes in acute brain slices of mice. We apply multiscale modeling to test the hypothesis that the temperature dependent [Ca^2+^]_i_ spiking is mainly caused by the increased activity of the sarcoendoplasmic reticulum ATPases (SERCAs) that remove Ca^2+^ from the cytosol into the endoplasmic reticulum. Quantitative comparison of experimental data with multiscale simulations supports the SERCA activity hypothesis. Further analysis of multiscale modeling and traditional rate equations indicates that the experimental observations are a spatial phenomenon where increasing pump strength leads to a decoupling of Ca^2+^ release sites and subsequently to vanishing [Ca^2+^]_i_ spikes.

## 1. Introduction

The human brain is by far the most complex organ; it hosts our consciences, is the source of our inspiration, and controls many of our vegetative functions. The extraordinary complexity of cellular interactions in terms of structural as well as dynamic properties enables the huge flexibility of our behavior and the amazing plasticity required for learning [1]. While traditional neuroscience has focused mainly on neurons for decades, the importance of the more abundant glia cells has only become evident more recently [2, 3].

Glia cells are generally known for their supportive function for neurons. The minority of glia cells are oligodendrocytes that myelinate axons [4]. More recently, oligodendrocytes were also found to essentially support axon integrity by metabolic coupling through monocarboxylate transporters (MCTs) [5, 6]. Microglia represent the macrophages of the brain and play an important role in immune responses as well as in modifying the neuronal network structure by synapse pruning [7]. The majority of glia cells are astrocytes that are commonly considered as metabolic supporters of neurons. On the one hand astrocytes regulate the blood flow in response to neuronal activity [8]. Astrocytes take up approximately 80% of the metabolized glucose from the blood and perform glycolysis [9]. According to the astrocyte-neuron lactate shuttle (ANLS) hypothesis [10], astrocytes generate lactate from pyruvate, the end product of glycolysis, by the lactate dehydrogenase (LDH) and export it into the extracellular space by MCTs. From the extracellular space, neurons take up the lactate and generate pyruvate by the reversible LDH reaction. The neuronal pyruvate is subsequently used as energy substrate for the TCA cycle in mitochondria to generate adenosine triphosphate (ATP) that is extensively needed to run the ion pumps in neurons to keep the large electrochemical potential across the plasma membrane. On the other hand, astrocytes play also an important function for neuronal proteostasis as they remove degraded material from the tissue either directly by intercellular transport towards the blood stream or by controlling the cerebral fluid flux [11].

Besides their supportive role in metabolism, astrocytes are now also known to modulate neuronal activity through active signaling mechanisms. Astrocytes are sealing up the dyadic cleft facilitating neurotransmitter diffusion and are essential for glutamate uptake and recycling [12]. Since astrocytes express also glutamate receptors, they can be stimulated by neuronal activity. Upon stimulation, astrocytes can secrete glutamate as well as ATP into the extracellular space including the dyadic cleft and thereby induce a local amplification mechanism. This neuron-astrocyte crosstalk has led to the picture of the tripartite synapse [13, 14].

The most predominant and studied signaling mechanism in astrocytes is inositol 1,4,5-trisphosphate (IP_3_) mediated Ca^2+^ signaling. In general, Ca^2+^ signaling is a versatile and universal second messenger that translates extracellular signals into intracellular responses like molecular motor activation or modulation of gene expression [15–17]. Within the IP_3_ pathway, extracellular agonists are detected by plasma membrane receptors. Upon binding, these G-protein coupled receptors activate the phospholipase C (PLC) that produces IP_3_ at the plasma membrane from which it diffuses into the cytosol. There IP_3_ can bind to IP_3_ receptors (IP_3_Rs) localized in the membrane of the endoplasmic reticulum (ER). When IP_3_ and Ca^2+^ are bound to the receptor, it can be open and Ca^2+^ is released from the ER into the cytosol due to the large concentration differences between these two compartments of typically several orders of magnitude. From the cytosol the released Ca^2+^ is moved back into the extracellular space by plasma membrane calcium ATPase (PMCA) and into the ER by sacroendoplasmic reticulum calcium ATPases (SERCAs).

The collection of the Ca^2+^ tool kit [18] can generate a wide spectrum of cellular responses ranging from long lasting high cytosolic Ca^2+^ levels ([Ca^2+^]_i_) to single [Ca^2+^]_i_ transients. The most common Ca^2+^ signals in astrocytes are repetitive cytosolic [Ca^2+^]_i_ transients often referred to as [Ca^2+^]_i_ oscillations [18] that are typically elicited by neurotransmitters and hormones but astrocytes do also exhibit spontaneous [Ca^2+^]_i_ spiking.

This oscillation-like behavior is rather common for many cell and stimulation types and has led to the general assumption that Ca^2+^ signaling is frequency coded [17]. This perspective is challenged by more recent studies including ours that have demonstrated that [Ca^2+^]_i_ spiking is indeed stochastic and therefore not truly oscillatory [19–21]. By systematic analysis of single cell [Ca^2+^]_i_ time courses for hundreds of cells of different cell types and stimulation antagonists, we found a preserved linear relation between the average period (*T*_av_) and the standard deviation (*σ*) of the inter-[Ca^2+^]_i_-spike intervals. Applying methods from the theory of stochastic processes, we have shown that this pathway specific linear *σ*-*T*_av_ relation characterizes the internal feedback strength [22] and represents an estimator for the information content [20, 23].

To understand the random signaling mechanism and underlying principles, we developed a computational multiscale model that takes into account the compartmentalization of the cell into cytosol and ER, the stochastic binding of signaling molecules to individual IP_3_Rs, their spatial organization into channel clusters, and the spatial diffusion of Ca^2+^ and Ca^2+^ binding buffers [27]. The enabled mechanistic simulations have shown that the experimental observations are in accordance with the implemented hierarchical stochastic system where single channel fluctuations are carried onto the level of the cell by diffusion mediated Ca^2+^ induced Ca^2+^ release (CICR). The modeling based insights on the functional robustness have guided further experiments that have revealed a fold change behavior in Ca^2+^ signaling of stimulated HEK cells and hepatocytes and were used to develop and validate a universal encoding relation [28].

The establishment of the signaling mechanism was driven to a large extent by experiments with astrocytes and microglia [21]. In particular the spontaneous activity of cultured astrocytes was exploited to obtain a baseline activity of the Ca^2+^ signaling machinery without potential antagonist induced feedback mechanisms. The astrocytic base level activity is characterized by a slope of 1 in the linear *σ*-*T*_av_ relation and corresponds to the theoretical minimal information gain. This is in accordance with the general assumption that stimulation should carry information that would correspond to a decreased slope of the *σ*-*T*_av_ relation and is observed in stimulated cells [20].

The spontaneous Ca^2+^ transients in astrocytes occur independently of neuronal activity but are modulated by neuronal function like induced epileptic activity [29]. The astrocytic Ca^2+^ increase can in turn be sensed by neurons because it has been correlated with release of neuroactive substances [30, 31]. Thereby astrocytes can release glutamate [32, 33], D-Serine [34, 35], ATP [36–39], chemokines [40], and nitric oxide (NO) [41]. The Ca^2+^ signal forms are rather heterogeneous and include random profiles and rhythmic oscillations as well as bursting activity.

Spontaneous [Ca^2+^]_i_ activity has been reported for astrocytes in culture [42–44] and acutely isolated slices of several brain regions [31, 45–47]. Spontaneous astrocytic Ca^2+^ signaling is modulated by pathological events such as those in reactive astrocytes surrounding stab wounds in the neocortex that lack spontaneous Ca^2+^ activity or astrocytes in the neocortex under epileptic conditions that show more spontaneously active astrocytes [45].

Despite all these insights, the common mechanism of this spontaneous activity is still not fully understood. In particular, the higher abundance of spontaneous [Ca^2+^]_i_ activity in cultured astrocytes compared to astrocytes within acute brain slices is only barely investigated [24]. We have shown in a comparison study that these differences can be explained to a large extent by the different temperatures at which the astrocytic [Ca^2+^]_i_ activity is typically measured. Here we revisit the experimental data and integrate it with our mechanistic multiscale modeling framework to quantitatively test our hypothesis on the molecular foundation of the temperature dependency.

## 2. Results

Motivated by the typically observed and reported significant differences in Ca^2+^ singling between cultured astrocytes and astrocytes in acute brain slices, we reconsidered the experimental workflow [24]. The most dramatic difference between the two experimental protocols is the different temperatures these two cell systems are typically measured at [21, 48]. In thermodynamics the general impact of temperature *T* on an individual reaction rate *k* is well approximated by the Arrhenius law by *k* = *Ae* ^−*E*_act_/*RT*^, where *A* denotes the approximate reaction rate, *E*_act_ describes the activation energy of the reaction, and *R* is the universal gas constant [49]. While the Arrhenius law describes the temperature dependency of an isolated reaction in a well-stirred reactor, the impact of temperature in more complex reaction systems may differ from the simple exponential relation. Alternatively it might be possible that the thermal energy in the measured temperature range is of the order of the activation energy, where the Arrhenius law shows a linear dependency. To explore if temperature could explain the Ca^2+^ signaling phenomenon, we summarize our careful analysis of temperature induced [Ca^2+^]_i_ dynamics changes and use here computational modeling to investigate potential reasons for this phenomenon.

### 2.1. Experiments Show Strong Temperature Dependency in Cortical and Cultured Astrocytes

In the experimental study [24] we used cortical slices obtained from P 10–P 12 mouse brains and cultured astrocytes. The temperature was either changed abruptly or smoothly either by changing the heating temperature of the water bath for the perfusion buffer or by switching from one perfusion buffer to another, respectively. Thereby the temperature was measured within the recording chamber close to the sample cover slip.

#### 2.1.1. Higher Temperatures Reversibly Suppress [Ca^2+^]_i_ Spiking Instantaneously

Starting from the standard protocols, we first measured Ca^2+^ activity at room temperature and separately at more physiological temperatures with Fluo4-AM loaded to the cells before (more detailed experimental description can be found in [24]). In cortical slices obtained from P 10–P 12 mouse brains measured at room temperature, astrocytes showed robust spontaneous signaling activity defined by long durations and large amplitudes of [Ca^2+^]_i_ spikes. In contrast, when slices were measured at physiological temperatures, only very few cells showed spontaneous activity.

**Figure 1:**
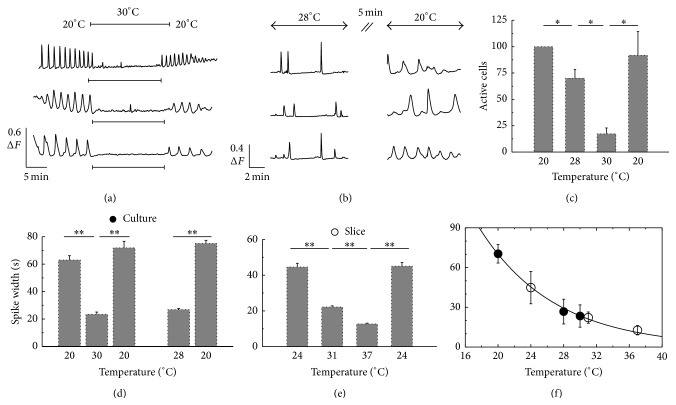
Summary of experimental observations on temperature dependent [Ca^2+^]_i_ activity [24]. (a) Fluorescence recordings from three individual astrocytes in three different experiments. The temperature was changed between 20°C and 30°C by using heated and nonheated perfusion buffer (indicated by the bar). Fluorescent amplitude Δ*F* is defined as the fluorescent temporal signal normalised by the initial signal strength. (b) [Ca^2+^]_i_ traces from three different cultured astrocytes at 28°C and 20°C. Note that the transient fluorescence changes have a smaller spike width and occur less frequently at higher temperature. The recordings were separated by 5 min. (c) Average number of cells which exhibited a [Ca^2+^]_i_ spike (average from four independent experiments). The number of responsive cells at 20°C was set to 100% and compared to the number of responsive cells at different temperatures or to the recovery at 20°C (bars indicate s.e.; ^∗^ *P* < 0.05). ((d), (e)). Average spike width of the fluorescent signals of all experiments obtained from cultured astrocytes (d) and in slices (e) determined at different temperatures as indicated (error bars indicate s.e.). (f) From the data the duration of the spike width is plotted as a function of temperature for astrocytes from culture (black dots) and brain slices (circles). The data points were fitted by *f*(*T*) = 620 s · exp⁡(−0.109*T*/°C) (^∗∗^ *P* < 0.005).

To compare this behavior with primary astrocytes cultured from mouse neocortex, we measured Ca^2+^ activity at room temperature (20°C) and then abruptly changed the perfusion buffer to one of 28°C or of 30°C in another setup. For these cultured astrocytes we observed a similar significant decrease of cells exhibiting Ca^2+^ signals already within the first minute after the temperature shift (Figure 1). Out of the active cells under 20°C (72 ± 24% of all cells in 4 independent experiments) more than a quarter of cells have not shown any [Ca^2+^]_i_ spikes under 28°C and under 30°C only 20% exhibited at least a single [Ca^2+^]_i_ spike. Remarkably, this dramatic decrease was fully reversible when perfusing the cells again with buffer at room temperature. Analogous results about the reversibility were also found in experiments with acute brain slices and the reversibility was independent of the experimental order of temperature changes.

The observed reduction in activity, starting at 28°C, was significantly smaller compared to recordings with the higher temperature of 30°C (Figure 1), indicating that between 28°C and 30°C is a threshold-like temperature with regard to [Ca^2+^]_i_ spike activity. The rather instantaneous response to temperature changes and the reversibility of the change in the activity pattern suggests that temperature is directly modifying the characteristic property of some molecular properties of the Ca^2+^ tool kit and not acting primarily on transcriptional regulation.

#### 2.1.2. Temperature Changes Modulate Signal Form of [Ca^2+^]_i_ Spike

Careful inspection of the time courses reveals also a strong temperature dependent on the duration and signal form of the Ca^2+^ transient in both cultured astrocytes and astrocytes in acute brain slices as shown in Figure 1. To quantify the effect on single [Ca^2+^]_i_-transients, we measured the spike width (SW) of individual Ca^2+^ transients by the full width half maximum (FWHM) method. Despite the cell specific heterogeneity, spike durations of the Ca^2+^ transients of astrocytes in acute brain slices decreased significantly when the temperature was raised from 24°C to 37°C. The decrease from 45 ± 17 s to 13 ± 4 s was fully reversible, since lowering the temperature back to 24°C increased the spike duration again to 45 ± 18 s (10 slices, 42 cells at *T*_24°C_  70 spikes; *T*_37°C_ 53 spikes; *T*_24°C_ 71 spikes). We additionally determined the spike duration at 31°C which averaged at 22 ± 5 s (9 slices, 29 cells at *T*_31°C_ 43 spikes) (Figure 1).

Within the analogous analysis of cultured astrocytes, we found that the spike duration changed from an average of 63 ± 18 s at 20°C to 21 ± 5 s at 30°C. This effect of temperature on spike duration was again reversible as spike duration increased again to 72 ± 20 s at 20°C after lowering the temperature from 30°C (4 independent experiments; at *T*_30°C_ 15 cells, 26 spikes; at *T*_20°C_ 30 cells, 56 spikes) (Figure 1). Within the other experimental setup, the average spike duration was 27 ± 6 s for 28°C as compared to 75 ± 16 s at 20°C (4 experiments; 30 cells at *T*_28°C_ 103 spikes; *T*_20°C_ 105 spikes).

The similarity of the signal form and particularly of the quantified spike widths of cells obtained from cultures and brain slices around 30°C points to temperature as the main reason for the typically reported differences in the signals. To analyze this behavior further we plotted the temperature dependence of the spike duration by pooling data from the two different experimental systems. The resulting data points can be fitted nicely by the exponential function *f*(*T*) = *ae* ^−*bT*^ with *a* = 620 s and *b* = 0.109/°C indicating a functional relation between spike width and temperature.

Within the experimental comparison study we also investigated the role of extracellular Ca^2+^ influx and intracellular Ca^2+^ release from intracellular stores. While Ca^2+^ free buffer has reduced the total number of responsive cells only slightly to 78 ± 34% (*n* = 13 slices, averaged cell number/slice = 31 ± 15), blocking SERCA pumps by loading 1 *μ*M thapsigargin for 15 min has abolished Ca^2+^ signals in more than 90% of cells at 31°C. Overall, these findings indicate a rapid change of the intracellular signaling mechanism. In particular, the astonishing reversibility of the Ca^2+^ activity and its signal forms when resetting the temperature to its previous value after tens of minutes suggests a molecular activity dependency on temperature.

### 2.2. Modeling the [Ca^2+^]_i_ Temperature Dependence by Modulated SERCA Activity

As previously speculated, the experimentally reported temperature dependent activity of the SERCA pumps [26, 50] may explain the observed changes in [Ca^2+^]_i_ signal forms. These studies report an increase of the pump strength with increasing temperature from 5°C to 38°C. Thus, the Ca^2+^ uptake at 5°C amounts only 5% of the uptake at 38°C. This can have a large effect on CICR that represents the spike generating mechanism because the IP_3_Rs are not homogeneously distributed within the cell but form channel clusters that are typically separated by 1 *μ*m or more. This separation may lead together with Ca^2+^ binding buffers and increasing SERCA activity to steeper gradients close to open channel clusters and subsequently to a weaker coupling between release sites and hence to a reduced CICR.

The data shown in Figure 1 support this perspective. The individual spike form is influenced by the stronger pumping as demonstrated experimentally by the decreased spike width for larger temperatures. Moreover, the spike widths of the two different cell types could be fitted nicely by one exponential function of the temperature. To test this SERCA based hypothesis we apply here our developed physiological multiscale model that is able to generate the whole spectrum of known [Ca^2+^]_i_ signals and was used to investigate the experimentally quantified stochastic nature of [Ca^2+^]_i_ spiking including the functional robustness of the *σ*-*T*_av_ relation [27].

#### 2.2.1. Multiscale Modeling Approach

Based on the observations that Ca^2+^ spiking is stochastic and obeys a nontrivial signaling signature, our multiscale model considers the molecular dynamics of individual IP_3_Rs and Ca^2+^ diffusion as well as buffer dynamics [27]. Our modeling strategy for this coupled system is based on separation of the two involved length scales. On the microscopic scale we use a detailed model for the channels [25]. Those will be open and close in a stochastic manner due to stochastic binding of the signaling molecules IP_3_ and Ca^2+^. The stochastic channel dynamics acts as a noisy source term that is driving the three-dimensional reaction diffusion system (RDS) representing the cytosol. Thereby the model also takes reactions of Ca^2+^ with buffer molecules like BAPTA or calmodulin and the SERCA pumps into account. From this perspective the cellular dynamics is given by an in general nonlinear reaction diffusion system that considers the cytosolic and lumenal dynamics by

(1a) ∂[Ca2+]∂t=Dc∇2[Ca2+]+σ~l+Pc(r,t) ·([E]−[Ca2+])−P~pCa2+2kd2+Ca2+2 +∑jkj−CaBj−kj+BjCa2+,

(1b) ∂[E]∂t=DE∇2[E]−γCa2+2kd2+Ca2+2σ~l+Pcr,tE−Ca2+         −P~pCa2+2kd2+Ca2+2 +∑kkk−EGk−kk+GkE,

(1c) $$\begin{array}{c} \frac{\partial \left[B_{j}\right]}{\partial t}=D_{B_{j}}\nabla^{2}\left[B_{j}\right]-k_{j}^{+}\left[B_{j}\right]\left[\text{C}{\text{a}}^{2+}\right]+k_{j}^{-}\left[\text{Ca}B_{j}\right], \end{array}$$

(1d) $$\begin{array}{c} \frac{\partial \left[G_{k}\right]}{\partial t}=D_{G_{k}}\nabla^{2}\left[G_{k}\right]-k_{k}^{+}\left[G_{k}\right]\left[E\right]+k_{k}^{-}\left[EG_{k}\right], \end{array}$$

where the first two equations describe the free Ca^2+^ concentration in the cytosol and the ER, respectively, and the other two equations correspond to a variety of cytosolic and lumenal buffers [*B*_*j*_] and [*G*_*k*_]. The first terms on the right hand side characterize diffusion with the individual diffusion coefficients *D*_*i*_. The following terms in (1a) and (1b) describe the leak flux (with permeability *σ*_*l*_) and release through channels (*P*_*c*_). The space dependence of *P*_*c*_(**r**_*c*_, *t*) reflects the location of channel clusters and the time dependent stochastic opening and closing of the channels. This time dependent source term is governed by stochastic simulation of the channel states. *γ* is the volume ratio *V*_cyt_/*V*_ER_. The third term results from Ca^2+^ uptake into the ER from the cytosol by SERCA pumps with a flux strength *P*_*p*_ and the SERCA dissociation constant *k*_*d*_. The remaining terms in (1a)–(1d) describe the reactions of Ca^2+^ with cytosolic and lumenal buffers [*B*_*k*_] and [*G*_*j*_], respectively, where *k* ^+^ denotes the capture rate and *k* ^−^ is the dissociation rate.

The cellular Ca^2+^ dynamics based on the CICR mechanism results from the local properties of the IP_3_R channels and their spatial coupling by Ca^2+^ diffusion that is influenced by buffer reactions and SERCA pump activity. Thus, the cellular Ca^2+^ signaling is determined by the channel states and corresponding release fluxes that shape the signals in dependence on the properties of the cellular diffusion. In turn, the resulting Ca^2+^ concentration profiles influence the channel opening behavior due to its dependence on the local Ca^2+^ concentration.

Standard simulation techniques for reaction diffusion systems like finite difference or finite element methods have to calculate the concentration profiles for the complete numerical mesh for convergence criteria. The large concentration gradients typically observed close to open channels require a fine spatial discretization that leads in particular in three spatial dimensions to large computational costs [51]. To circumvent this restriction and enable simulations of biological relevant time courses, we developed an analytic solution of the reaction diffusion equations in terms of coupled Green's function that allows for calculating [Ca^2+^]_i_ at specific spatial positions only such as channel cluster locations. These local concentrations subsequently determine potential changes in the stochastic channel dynamics leading to modified source terms in the reaction diffusion system.

In order to solve the reaction diffusion system analytically we restrict the system to one mobile and one immobile buffer, introduce dimensionless variables, and linearize the corresponding equations [27]. To obtain an analytic solution of the resulting reaction diffusion system we have to determine the geometry and boundary conditions. As a natural choice we use a spherical geometry and, motivated by the observation that astrocytic Ca^2+^ signals do not change significantly in Ca^2+^ free medium [24], no-flux boundary condition that reflects the properties of the plasma membrane for our in silico cell. With these specifications we could develop the analytic solution by coupled Green's functions [52, 53] describing Ca^2+^ and buffer dynamics where open channels correspond to source terms. For mathematical details of the implemented Green's cell algorithm (GCA) see [27] where we have shown how microscopic channel fluctuations are transmitted onto the level of the cell by diffusion thereby matching the experimental findings on cell variability and buffer influence.

**Figure 2:**
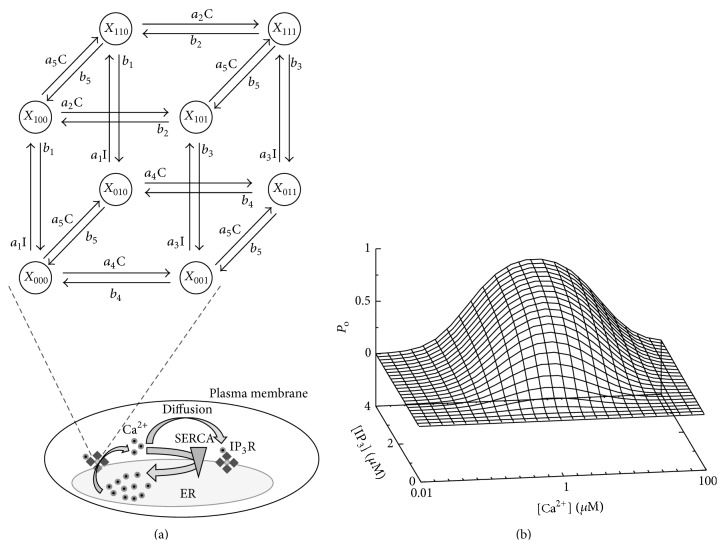
CICR is based on the nonlinear open probability of IP_3_R. (a) Scheme of the DeYoung-Keizer model for a single subunit. A subunit is active, if IP_3_ (I) is bound and Ca^2+^ (C) is only bound to the activating site, that is, in state *X*_110_. A channel opens if at least 3 of its 4 subunits are active. Open IP_3_Rs release Ca^2+^ that can diffuse to other IP_3_R clusters and activate the channels there as well, eventually leading to cell wide Ca^2+^ release and generating a [Ca^2+^]_i_ spike. From the cytosol Ca^2+^ is pumped back into the ER by SERCA that decreases diffusion. (b) From the Markov chain model of a single subunit the non-Markovian stationary open probability *P*_*o*_ of a channel can be calculated in dependence on Ca^2+^ and IP_3_ concentration [25].

For the IP_3_R description within the GCA, we use the DeYoung-Keizer model (DKM) that assumes three binding sites for each of the four subunits per channel [54]. Each of the three binding sites can be free or occupied leading to 2^3^ different states *X*_*ijk*_ and 12 possible transitions in dependence on the Ca^2+^ concentration C and IP_3_ concentration I that can be visualized on a cube as shown in Figure 2(a). A subunit is active in the state *X*_110_ only where the first index equals one if IP_3_ is bound, the second index corresponds to Ca^2+^ binding to an activating site, and the last index describes the occupation of the dominant inhibiting Ca^2+^ binding site. The activating binding sites for Ca^2+^ have a higher affinity compared to the dominant inhibiting site. This is a minimal choice to generate the nonlinearity in Ca^2+^, which is the source of the bell shaped open probability (Figure 2(b)) and the basis of CICR [25].

#### 2.2.2. Modeling the Impact of SERCA Pump Strength on Ca^2+^ Dynamics

Here we use our validated GCA implementation to test the hypothesis on the temperature dependent SERCA activity as a main reason for the observed differences in [Ca^2+^]_i_ spiking. For the simulations, we use physiological cellular diffusion parameters listed in Table 1. Our in silico cell has 31 randomly distributed IP_3_R clusters each of them having a random number of channels between 2 and 16, yielding 258 channels in total. The clusters are separated by at least 1.5 *μ*m and only located within a sphere with 80% of the cell radius *R* to guarantee convergence of Green's function representation. To test the SERCA hypothesis, this prototype of a cell was simulated with different pump strengths *P*_*p*_.

**Figure 3:**
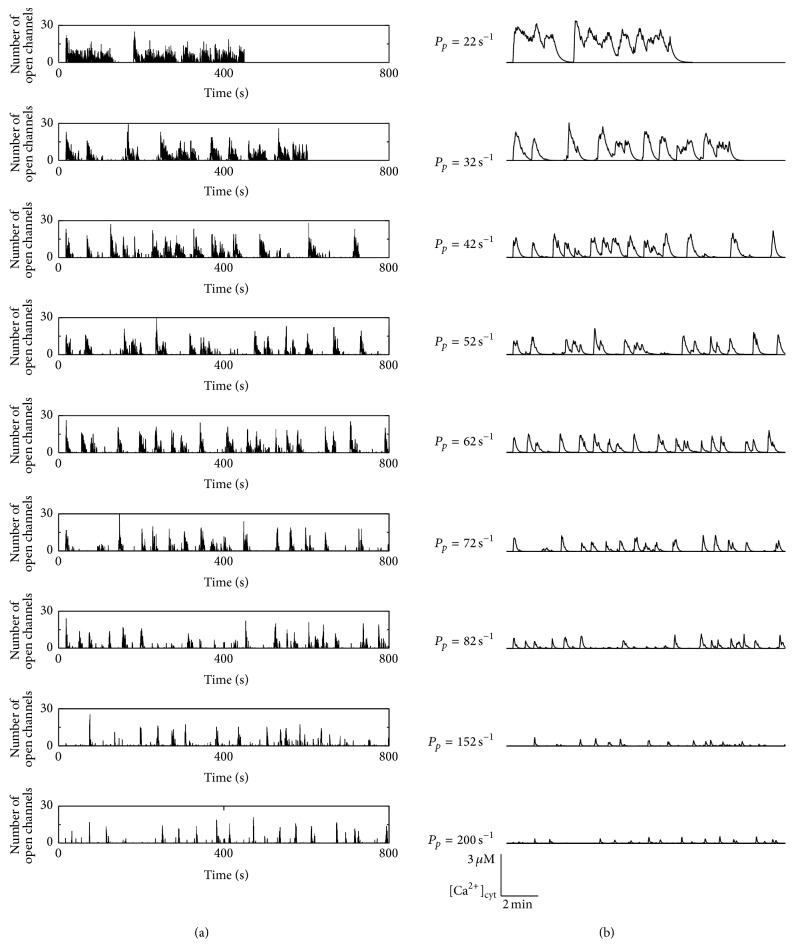
Dependence of Ca^2+^ signals on the pump strength. The temperature dependent activity of SERCA leads to higher pump rates *P*_*p*_ for higher temperatures. This influences the Ca^2+^ signals as shown by the number of open channels (a) and the corresponding Ca^2+^ concentrations (b). For the two smallest strengths the simulations ended before the shown real time of 800 s caused by run time restrictions on the compute cluster.

Figure 3 shows the cell wide dynamics, where panel (a) exhibits the number of open channels and panel (b) shows the resulting cytosolic Ca^2+^ concentration for pump strengths *P*_*p*_ varying between 22 s^−1^ and 200 s^−1^ what is around the estimated physiological reference value of 86 s^−1^ [55]. A first conspicuous property shown in panel (a) is that the amplitudes of open channels during a spike are rather similar and vary between 25 and 30, rather independently of the pump strengths *P*_*p*_. Nevertheless, *P*_*p*_ has a huge influence on the time course.

For low pump strengths, Ca^2+^ is removed too slowly from the cytosol for a coordinated signal. During the initial activation from base level the amplitude of open channels becomes maximal and is limited by inhibition according to the channel properties explained in Figure 2. When a channel recovers from inhibition due to the stochastic unbinding of Ca^2+^ from a dominantly inhibiting site of a subunit, the cytosolic Ca^2+^ concentration is still in the Ca^2+^ concentration range where the higher affinity for the Ca^2+^ activating binding site leads to a large open probability shown in Figure 2(b). Subsequently, the channel will open again, increase the local Ca^2+^ concentration, and reinduce channel inhibition. Repeating this scenario, the channels will mainly switch between activation and inhibition back and forth where a rather constant proportion of up to 50% of subunits are inhibited until randomly no reactivation occurs and the cell is reaching its resting state again. This permanent switching between activation and inhibition leads to the observed plateaus for the two lowest pump strengths.

This behavior is also reflected by the resulting cytosolic Ca^2+^ concentrations. For *P*_*p*_ = 22 s^−1^, the cell exhibits a plateau response with some superimposed [Ca^2+^]_i_ spiking, which are often observed in experiments with astrocytes from acute brain slices [24, 56–58]. For *P*_*p*_ = 32 s^−1^, the shorter plateaus of open channels are translated into a more oscillation-like behavior of the cytosolic Ca^2+^ concentration exhibiting rather diverse spike amplitudes. The concentration is in a similar range as the cell with the lower pump strength, indicating a regime where local channel inhibition and spatial coupling determined by pumps have an equal influence on the cellular dynamics.

For increasing *P*_*p*_, the cells exhibit an even more spiking like behavior since long lasting channel activity is absent, and therefore [Ca^2+^]_i_ shows more pronounced peaks. This can be again explained by the interplay of inhibition and Ca^2+^ removal. After a spike has occurred and most channels are inhibited, the SERCAs decrease the cytosolic Ca^2+^ concentration sufficiently fast leading to base level [Ca^2+^]_i_ when channels recover from inhibition. Subsequently, the cell relaxes to its resting level, from which it can be activated again by CICR, that is, by triggering opening of a channel cluster. For very large pump strengths, the spikes of the number of open channels have still a similar height to those for smaller *P*_*p*_, but their widths decrease drastically. This induces low and slim peaks of the cytosolic Ca^2+^ concentration.

So far, the simulations seem to support the hypothesis of the decreased SERCA activity leading to an increased [Ca^2+^]_i_ activity at lower temperatures. In terms of the experimental findings, a temperature increase in experiments and observed changes in the Ca^2+^ signals correspond to an increase of the pump rate from the region from 42 s^−1^ to 62 s^−1^ to 152  s^−1^ or higher.

**Figure 4:**
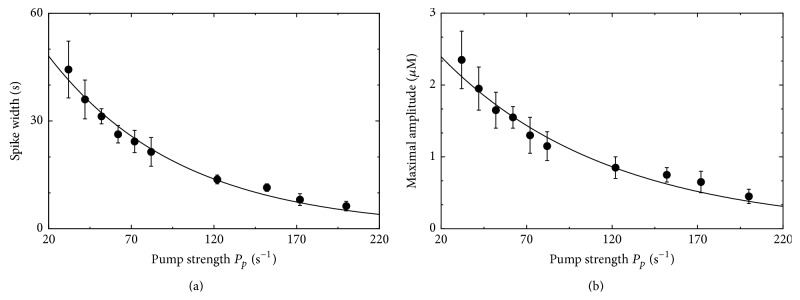
Spike width and amplitude in dependence on the pump strength for the Ca^2+^ signal shown in Figure 3. (a) The spike width exhibits an exponential dependence on *P*_*p*_. This is in good agreement with the experimental findings in Figure 1. (b) Also the amplitude dependence on *P*_*p*_ obeys an exponential relation (error bars denote s.e.).

For a further detailed analysis, we determine the spike widths of the simulated cytosolic Ca^2+^ peaks as done for the experimental data in Figure 1.  Figure 4(a) exhibits the dependence of the spike width on the pump strength *P*_*p*_. The analysis reveals an exponential dependence on the pump rate *P*_*p*_ as shown by the line defined by SW = 61 s · *e* ^−0.012 s·*P*_*p*_^. At second glance we observe that the spike width becomes most regular for *P*_*p*_ = 62 s^−1^ as depicted by the small error bar that exhibits the smallest relative variation even compared to the smaller error bars for large *P*_*p*_. This might indicate an optimal dynamical regime.

An analogue behavior can be found for the average amplitudes in Figure 4(b). The dependence on *P*_*p*_ can be fitted by the exponential relation 2.9 · *e* ^−0.011 s·*P*_*p*_^, and again the mean amplitude for *P*_*p*_ = 62 s^−1^ exhibits the smallest relative variations. The amplitude behavior obtained from simulations has a similar trend like those seen in the experiments but contradicts former theoretical results where the amplitude stayed constant [59].

**Figure 5:**
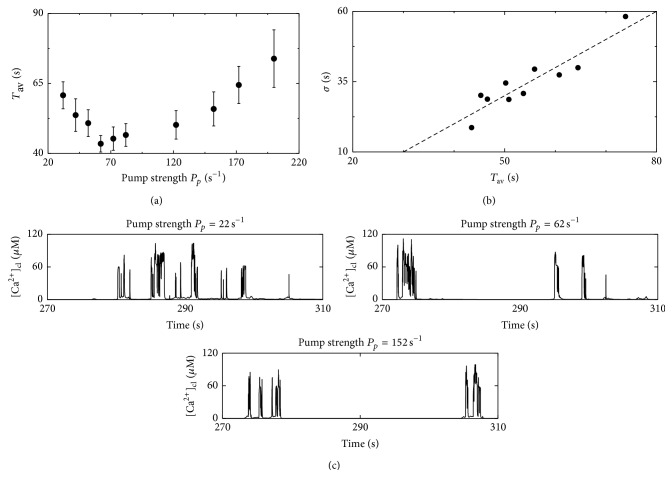
Dependence of the mean period *T*_av_ on the pump strength *P*_*p*_. (a) *T*_av_ exhibits a nonlinear dependence on the pump strength with a minimal mean period of 42 s for *P*_*p*_ = 62 s^−1^. This might illuminate a possible control mechanism cells can use to tune their oscillations. (b) The *σ*-*T*_av_ relation exhibits a population slope close to one and a deterministic time of approximately 20 s. (c) The height of the local Ca^2+^ concentration [Ca^2+^] close to a cluster is hardly affected by *P*_*p*_ as demonstrated by the representative example of the same cluster in different simulations using different pump rates. Hence, the differences in the cytosolic Ca^2+^ oscillations in Figure 3(b) are spatially induced.

To analyze the potential optimal regime, we determine the oscillation characteristics *T*_av_ and *σ*. Indeed, *T*_av_ exhibits a minimum of 42 s for a pump rate of 62 s^−1^ as shown in Figure 5(a). Panel (b) displays the dependence of *σ* on *T*_av_ showing a slope close to one as in earlier experiments and simulations and indicates a deterministic time of 20 s. The spread of the data points is exclusively caused by the different pump strengths. Similar simulations with smaller buffer concentrations have shown an analogue behavior with a shift of the minimum of *T*_av_ to larger *P*_*p*_. This is in line with our consideration, since a lower buffer concentration leads to an increased spatial coupling that can be compensated by higher pump rates.

For an additional test of our conception based on the spatial coupling of release sites, we analyze the local Ca^2+^ concentrations at channel clusters in Figure 5(c). For all pump strengths the concentration peaks are in the range of 100 *μ*M independently of *P*_*p*_. Hence, the different cell wide cytosolic Ca^2+^ signals shown in Figure 3(b) are not caused by different local properties but are caused by the spatial coupling of release sites. Thus, the results of the temperature experiments are consistent with the hypothesized spatial control mechanism.

#### 2.2.3. Rate Equation Based Modeling

To further investigate the role of the spatial aspect in Ca^2+^ signaling, we model the effect of SERCA activity on the Ca^2+^ profile by a traditional rate equation model implemented by ordinary differential equations (ODEs). These types of models do not consider any spatial aspects but treat the cell as a well-stirred homogeneous entity and were extensively used in the establishment of the Ca^2+^ signaling mechanism [60, 61]. A variety of models have been proposed and applied to diverse aspects of Ca^2+^ signaling where the common mechanism is Ca^2+^ cycling between the ER and the cytosol. Here we use a model based on previous studies which reflects the main Ca^2+^ fluxes according to Figure 2 as well as Ca^2+^ buffering by mitochondria. The governing rate equations for the cytosolic and ER Ca^2+^ concentration read

(2a) $$\begin{array}{c} \frac{d{\text{Ca}}_{c}}{dt}=f_{\text{cyt}}\left(J_{\text{e}\;\text{xt}}+\Delta J_{\text{mito}}+J_{\text{release}}+J_{\text{leak}}-J_{\text{serca}}-J_{\text{pmca}}\right), \end{array}$$

(2b) $$\begin{array}{c} \frac{d{\text{Ca}}_{\text{er}}}{dt}=f_{\text{er}}\gamma^{-1}\left(J_{\text{serca}}-J_{\text{release}}-J_{\text{leak}}\right), \end{array}$$

where *f*_cyt_ and *f*_er_ are the fractions of free Ca^2+^ within the cytosol and the ER [62], *γ* is again the volume ratio between cytosol and ER, Δ denotes the mitochondria to cytosol volume ratio, and the corresponding fluxes *J* reflect typical physiological characteristics. The 2 most important fluxes are the Ca^2+^ release from the ER by IP_3_R (*J*_release_) and the SERCA mediated Ca^2+^ removal from the cytosol into the ER (*J*_serca_) given by

(3a) Jrelease=PrIP32p12+IP32Cac2p22+Cac2 ·p34p44+Cac4Caer−Cac,

(3b) $$\begin{array}{c} J_{\text{serca}}=P_{p}\left(\frac{{\text{Ca}}_{c}^{2}}{p_{5}^{2}+{\text{Ca}}_{c}^{2}}\right), \end{array}$$

respectively. The dominant flux for the [Ca^2+^]_i_ spiking is the nonlinear release flux inducing the CICR by the bell shaped dependence on the Ca^2+^ concentration [63] here modeled in analogy to previous approaches [64]. The form of the complementary SERCA mediated Ca^2+^ flux into the ER (*J*_serca_) reflects the fact that two Ca^2+^ ions are pumped into the ER by the use of one ATP molecule where the ATP concentration is assumed to be constant. The remaining fluxes are given in Table 2 where *J*_e xt_ and *J*_mito_ describe Ca^2+^ flux from extracellular space and mitochondria, respectively. *J*_leak_ corresponds to the Ca^2+^ leakage from ER and *J*_pmca_ is the flux into the extracellular space through plasma membrane Ca^2+^ ATPases. The corresponding parameters *p*_*i*_ are also listed in Table 2.

**Figure 6:**
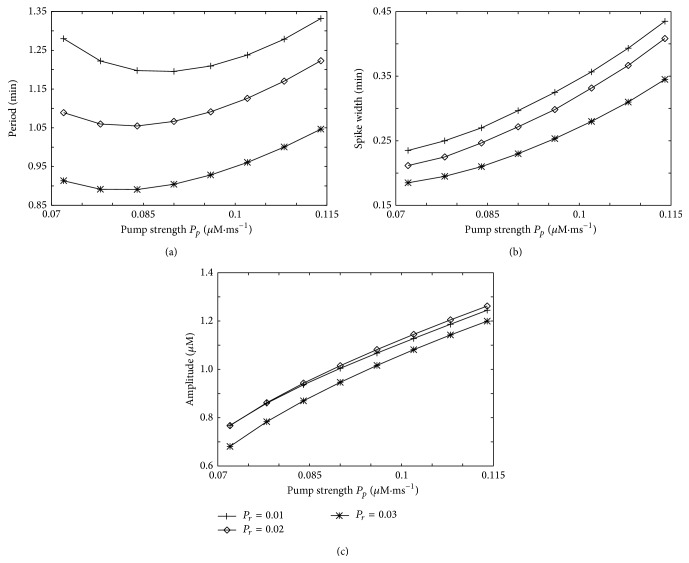
Cell characteristics in the rate equation model. Dependence of the period (a), amplitude (b), and spike width (c) on the SERCA pump strength *P*_*p*_ for three different Ca^2+^ release rates *P*_*r*_ from IP_3_ channels.

To investigate the influence of SERCA pump strength on Ca^2+^ dynamics, we simulate the ODEs for a wide range of pump strength *P*_*p*_ and three different values of the Ca^2+^ release flux (*P*_*r*_) from IP_3_R channels and analyze the spike characteristics (Figure 6). This parameter scan shows that the Ca^2+^ period within the rate equation model exhibits a similar behavior as the spatial GCA simulations in terms of a minimal period in dependence on the pump strength (Figure 6(a)). While this finding is not in clear favor for the perspective of spatial coupling of release sites as the [Ca^2+^]_i_ spike generating mechanism, the results obtained with the rate equation model given by (2a) and (2b) about the spike width and amplitude exhibit a different behavior than the experimental data.

In the ODE model a higher SERCA pump rate causes more Ca^2+^ within the ER and also a higher Ca^2+^ release into the cytosol because IP_3_R flux is controlled to a large proportion by the concentration difference and thus by the ER concentration. A high SERCA pump rate leads to a larger spike width as observed in Figure 6(b). These higher fluxes in and out of the ER also yield higher spike amplitudes (Figure 6(c)). Both observations are in contrast to the experimental observations and the GCA simulations, potentially indicating the fact that a spatial distribution of channels is a necessity for the experimentally observed SERCA pump effects.

## 3. Discussion

Previous experimental findings have shown a clear temperature dependency of Ca^2+^ signals in astrocytes and indicated the different temperatures at which cultured astrocytes and astrocytes in acute brain slices are typically measured as a potential explanation for the reported differences in Ca^2+^ signaling [24]. Based on these observation and experimental studies reporting a temperature dependent SERCA activity, we used here multiscale modeling to investigate if this activity change may explain the experimental finding.

For this purpose we simulated in silico cells with our GCA implementation that allows for controlling all physiological parameters and obtaining mechanistic insights. From the activity patterns of identical in silico cells that only differ in the SERCA activity shown in Figure 3, we can directly conclude that the interplay of the IP_3_R properties with respect to CICR and inhibition together with the SERCA pumps can generate a wide spectrum of cellular Ca^2+^ signals. In particular, [Ca^2+^]_i_ spiking occurs for an intermediate pump rate where Ca^2+^ is removed fast enough from the cytosol to prevent too frequent repetitive stimulation but where the spatial coupling between the release sites is preserved to allow for cell wide coordinated signals.

As a more quantitative analysis we analyzed the spike width in the simulation data in analogy to the experimental findings where we found that the experimental spike width followed an exponential dependency on temperature independently if the width was determined from cultured astrocytes or from astrocytes in acute brain slices (Figure 1). Remarkably, we obtained again an exponential dependency of the spike width from the simulations as shown in Figure 4. The modeling based exponential relation does not depend explicitly on the temperature but depends on the pump strength *P*_*p*_ that was varied in simulations. From the obtained two exponential dependencies of the spike width on the temperature in experiments *g*(*T*) = *a*exp⁡[−*bT*] and on the SERCA activity in simulations *f*(*P*_*p*_) = *α*exp⁡[−*βP*_*p*_] we can infer the relation between these two quantities by setting *g*(*T*) ∝ *f*(*P*_*p*_) as

(4) $$\begin{array}{c} P_{p}\left(T\right)=\frac{b}{\beta }T+\frac{\ln\left(\alpha /a\right)}{\beta }. \end{array}$$

**Figure 7:**
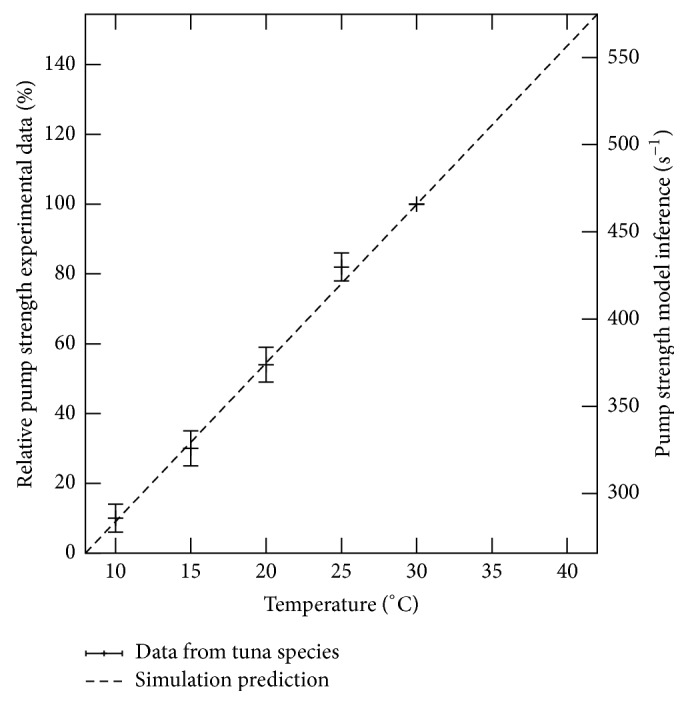
SERCA pump activity depends linearly on the temperature as shown independently by our model inference (line) and by experimental data from tuna species (data points from [26], Figure 2(b)). For the experimental data points the pump strength is set to 100% at 30°C and used for normalisation for the lower temperatures leading to relative activity values shown on the left vertical axis. As a comparison the inference from our simulation (4) is shown by the straight line with the absolute values given on the right vertical axis.

This relation predicts a linear relation between the SERCA activity *P*_*p*_ and the temperature *T* as shown in Figure 7 where the model inference is shown by the line. Note that the proportional assumption can lead to an additional scaling factor in the second term of (4) but not in the first one that describes the slope of the relation. A linear relation was also measured experimentally in tuna by others [26]. Within this study no absolute values of the pump strength were measured but relative changes for different temperatures compared to a reference at 30°C were determined as shown in Figure 7 by the data points. The qualitative agreement between the directly measured relative changes (data points in Figure 7) and the spike width based model inference (line in Figure 7) is another strong evidence that modified SERCA activity may induce the change in astrocytic Ca^2+^ signals at different temperatures.

It is worth emphasizing that we varied in our multiscale simulations the pump strength motivated by the experimentally reported temperature dependent SERCA activity but did not include any specific relation. From the unbiased parameter scan on *P*_*p*_ we first observed the general tendency of less coordinated Ca^2+^ signals for higher temperature in accordance with the experimental data. More strikingly, we found an exponential dependency on *P*_*p*_ of the spike width in the simulation data in analogy to the exponential dependency of the spike width on the temperature in experiments. The exponential relation was not included explicitly within the model but followed naturally from the physiological simulations indicating the importance of the spatial signal organization. With the two obtained exponential relations for the spike width *g*(*T*) from experiments and *f*(*P*_*p*_) from simulations, we then inferred the linear relation between SERCA activity *P*_*p*_ and temperature *T* shown by the line in Figure 7. This inferred linear relation is in great qualitative accordance with the previous directly measured temperature dependency of the SERCA activity shown by the data dots in Figure 7. While the previous study has motivated the modeling strategy of modulating the pump strength *P*_*p*_, no specific relation has been assumed but is only caused by the spatial signal mechanism.

Strikingly, the two independently obtained relations for the SERCA pump activity both exhibit a linear dependency on the temperature questioning the applicability of the Arrhenius law in this case.

The finding either indicates an activation energy *E*_act_ in the order of the thermal energy *RT* or a more complex reaction system than what the Arrhenius law is considering. While the activation energy of SERCA is larger than the thermal energy as indicated by the use of ATP for the pump reaction, the underlying effective reaction scheme is more complex. The pump reaction consists of several (potentially interdependent) binding steps of Ca^2+^ and ATP to the SERCA protein. Since each of the binding and unbinding processes will be affected by temperature changes, the resulting reaction may exhibit already different characteristics from the collision of free particles assumed in Arrhenius law. More importantly, we determine here the cell wide averaged SERCA activity. The modulated SERCA activity is changing the spatial coupling between the release sites which leads to a modified hierarchical signal organisation by changing diffusion properties within the diffusion mediated CICR mechanism. This renders the reaction scheme even more complex and may be the main reason for the deviation from Arrhenius law.

When comparing the different scales for the pump strength-temperature dependence in Figure 7, it becomes obvious that the used SERCA activity parameters *P*_*p*_ in the simulations were smaller than those we would expect from the inference. Furthermore, the slope of the relation from inference is twice as large as the linear fit to the experimental data. This quantitative discrepancy is potentially caused by slightly different spatial characteristic like Ca^2+^ buffer content and physiologic settings of the cells. Nevertheless, the integrative approach shows how molecular properties can be characterized from global cell behavior by mechanistic modeling.

To further evaluate the spatial characteristic of the phenomenon, we were also analyzing local concentrations at channel clusters. Their amplitudes were rather unaffected by the pump strength but the collective IP_3_R activity was significant modulated. This is a further evidence that it is the cell level that shapes the [Ca^2+^]_i_ signals and not local properties. In line with these findings, we found that the rate equation based model (2a) and (2b) is unable to recapitulate the experimental finding when modulating the pump activity. This model approach neglects the spatial scale and thus the spatial coupling between release sites. The failure to model the experimental observations can be also seen as an indirect support for the spatial characteristic of Ca^2+^ signals. In general, the observed experimental behaviors might be also obtained by specific realizations of rate equation models that might consider slightly different flux definitions or integrate further pathways like ATP consumption by SERCA pumps and facilitated ATP production by Ca^2+^ triggered mitochondrial activity. These models are typically rather focused on specific scenarios and often not able to generate the wide spectrum of observed Ca^2+^ signals. In contrast, we have shown that the multiscale approach of the GCA is able to generate all these experimental observations in dependence on reasonable physiological parameters [27].

Interestingly, we found in the multiscale GCA simulations a regime of optimal signaling in terms of regularity of Ca^2+^ spikes. The nonlinear dependence of *T*_av_ on the pump strength might indicate a possible control mechanism. A recent study [65] has reported how frequency modulation may control gene regulation. If we therefore assume that the ability to spike and to use frequency coding is the purpose of the Ca^2+^ signaling pathway, cells can control this behavior by the expression level of SERCA. From our findings here, we would expect a negative feedback of SERCA expression on fast oscillations and a positive feedback on slow oscillations with high concentration peaks and plateaus.

The impact of temperature on Ca^2+^ signaling is probably not an astrocyte-specific phenomenon. In cardiac muscle the frequency of Ca^2+^ spikes decreases dramatically at 37°C compared with 22°C. In other cell types like in rabbit renal tubules, hepatocytes, and parenchymal and endothelial cells acute hypothermia affects the intracellular Ca^2+^ homeostasis and goes along with a rise in cytosolic Ca^2+^ levels [66–68]. Due to the wide distribution of SERCA and the universality of Ca^2+^ signaling, this mechanism might be used in many temperature sensitive cellular processes, which could make SERCA a key temperature sensor.

## Figures and Tables

**Table 1:** Physiologic parameters of the in silico cell used in simulations. Note that the GCA uses a linearized reaction diffusion system leading to rates for *σ*_*l*_ and *P*_*p*_.

Physiological cell parameter values for GCA simulations		
*R*	10 *μ*m	Cell radius
*γ*	5	Volume ratio between cytosol and ER
*D*_Ca_	220 *μ*m^2^/s	Diffusion coefficient of cytosolic Ca^2+^
*D*_*E*_	70 *μ*m^2^/s	Diffusion coefficient of lumenal Ca^2+^
*D*_*B*_	95 *μ*m^2^/s	Diffusion coefficient of mobile buffer
[Ca^2+^]_0_	55 nM	Cytosolic Ca^2+^ base level
[E]_0_	800 *μ*M	Endoplasmic reticulum Ca^2+^ base level
[IP_3_]	80 *μ*M	IP_3_ concentration
[*B*]_*T*_	52 *μ*M	Total mobile buffer concentration (EGTA)
*k*_*B*_^+^	0.3 (*μ*Ms)^−1^	On rate of the mobile buffer
*k*_*B*_^−^	1.5 s^−1^	Dissociation rate of the mobile buffer
[*B*_*i*_]_*T*_	30 *μ*M	Total immobile buffer concentration
*k*_*B*_*i*__^+^	600 (*μ*Ms)^−1^	On rate of the immobile buffer
*k*_*B*_*i*__^−^	100 s^−1^	Dissociation rate of the immobile buffer
*σ*_*l*_	≈0.01 s^−1^	Leak flux constant given by varied *P*_*p*_, [Ca^2+^]_0_ and [E]_0_

**Table 2:** Physiologic parameters of the in silico cell used for the rate equation simulations.

Additional flux definitions of the full rate equation model			
Sodium-Ca^2+^ exchanger mediated flux		$J_{\text{NaCa}}=p_{5}\frac{\text{C}{\text{a}}_{m}}{\text{C}{\text{a}}_{m}+p_{6}}\frac{p_{7}^{2}}{p_{8}^{2}+\text{C}{\text{a}}_{c}^{2}}$	
Mitochondrial unitransporter mediated flux		$J_{\text{uni}}=p_{9}\frac{\text{C}{\text{a}}_{c}^{2}}{p_{10}^{2}+\text{C}{\text{a}}_{c}^{2}}$	
Effective mitochondrial flux		*J*_mito_ = *J*_NaCa_ − *J*_uni_	
Leak flux through ER membrane		*J*_leak_ = *p*_11_(Ca_er_ − Ca_*c*_)	
Influx from external space		*J*_*ext*_ = *J*_ext,bas_ − (*p*_12_Ca_er_ + *p*_13_Ca_*c*_)	
PMCA mediated flux		$J_{\text{pmca}}=p_{14}\frac{\text{C}{\text{a}}_{c}^{2}}{p_{15}^{2}+\text{C}{\text{a}}_{c}^{2}}$	

Parameter values for the rate equation calcium model			

P_*r*_ = 0.03 ms^−1^	p_1_ = 0.5 *μ*M	p_2_ = 0.9 *μ*M	p_3_ = 1.3 *μ*M
p_4_ = 0.2 *μ*M	P_*p*_ = 0.06 *μ*M ms^−1^	p_5_ = 10 *μ*M ms^−1^	p_6_ = 0.6 *μ*M
p_7_= 1 *μ*M	p_8_ = 10 *μ*M	p_9_ = 0.2 *μ*M ms^−1^	p_10_ = 1 *μ*M
p_11_ = 10^−3^ ms^−1^	p_12_ = 3 · 10^−6^ ms^−1^	p_13_ = 15 · 10^−6^ ms^−1^	p_14_ = 0.01 *μ*M ms^−1^
p_15_ = 0.7 *μ*M	IP_3_ = 0.4 *μ*M	f_cyt_ = 0.015	f_er_ = 0.5
J_ext,bas_ = 3.5 · 10^−3^ *μ*M ms^−1^	Δ = 0.07	*γ* ^−1^ = 30	

## References

[1] Cherniak C. (1990). The bounded brain: toward quantitative neuroanatomy. *Journal of Cognitive Neuroscience*.

[2] Allen N. J., Barres B. A. (2009). Neuroscience: Glia—more than just brain glue. *Nature*.

[3] Azevedo F. A. C., Carvalho L. R. B., Grinberg L. T. (2009). Equal numbers of neuronal and nonneuronal cells make the human brain an isometrically scaled-up primate brain. *Journal of Comparative Neurology*.

[4] Baumann N., Pham-Dinh D. (2001). Biology of oligodendrocyte and myelin in the mammalian central nervous system. *Physiological Reviews*.

[5] Fünfschilling U., Supplie L. M., Mahad D. (2012). Glycolytic oligodendrocytes maintain myelin and long-term axonal integrity. *Nature*.

[6] Lee Y., Morrison B. M., Li Y. (2012). Oligodendroglia metabolically support axons and contribute to neurodegeneration. *Nature*.

[7] Kreutzberg G. W. (1996). Microglia: a sensor for pathological events in the CNS. *Trends in Neurosciences*.

[8] Takano T., Tian G.-F., Peng W. (2006). Astrocyte-mediated control of cerebral blood flow. *Nature Neuroscience*.

[9] Pellerin L., Pellegri G., Bittar P. G. (1998). Evidence supporting the existence of an activity-dependent astrocyte-neuron lactate shuttle. *Developmental Neuroscience*.

[10] Hertz L. (2004). The astrocyte-neuron lactate shuttle: a challenge of a challenge. *Journal of Cerebral Blood Flow and Metabolism*.

[11] Nedergaard M. (2013). Garbage truck of the brain. *Science*.

[12] Allen N. J. (2014). Synaptic plasticity: astrocytes wrap it up. *Current Biology*.

[13] Parri H. R., Gould T. M., Crunelli V. (2001). Spontaneous astrocytic Ca^2+^ oscillations in situ drive NMDAR-mediated neuronal excitation. *Nature Neuroscience*.

[14] Halassa M. M., Fellin T., Haydon P. G. (2007). The tripartite synapse: roles for gliotransmission in health and disease. *Trends in Molecular Medicine*.

[15] Berridge M. J. (1987). Inositol trisphosphate and diacylglycerol: two interacting second messengers. *Annual Review of Biochemistry*.

[16] Berridge M. J. (1993). Inositol trisphosphate and calcium signalling. *Nature*.

[17] Berridge M. J., Lipp P., Bootman M. D. (2000). The versatility and universality of calcium signalling. *Nature Reviews Molecular Cell Biology*.

[18] Bootman M. D., Berridge M. J. (1995). The elemental principles of calcium signaling. *Cell*.

[19] Dupont G., Abou-Lovergne A., Combettes L. (2008). Stochastic aspects of oscillatory Ca^2+^ dynamics in hepatocytes. *Biophysical Journal*.

[20] Skupin A., Falcke M. (2008). Statistical properties and information content of Ca2^+^ oscillations. *Genome Informatics*.

[21] Skupin A., Kettenmann H., Winkler U. (2008). How does intracellular Ca^2+^ oscillate: by chance or by the clock?. *Biophysical Journal*.

[22] Skupin A., Falcke M. (2010). Statistical analysis of calcium oscillations. *European Physical Journal: Special Topics*.

[23] Skupin A., Falcke M. (2009). From puffs to global Ca^2+^ signals: how molecular properties shape global signals. *Chaos*.

[24] Schipke C. G., Heidemann A., Skupin A., Peters O., Falcke M., Kettenmann H. (2008). Temperature and nitric oxide control spontaneous calcium transients in astrocytes. *Cell Calcium*.

[25] Christian N., Skupin A., Morante S., Jansen K., Rossi G., Ebenhöh O. (2014). Mesoscopic behavior from microscopic Markov dynamics and its application to calcium release channels. *Journal of Theoretical Biology*.

[26] Landeira-Fernandez A. M., Morrissette J. M., Blank J. M., Block B. A. (2004). Temperature dependence of the Ca2+-atpase (serca2) in the ventricles of tuna and mackerel. *The American Journal of Physiology—Regulatory Integrative and Comparative Physiology*.

[27] Skupin A., Kettenmann H., Falcke M. (2010). Calcium signals driven by single channel noise. *PLoS Computational Biology*.

[28] Thurley K., Tovey S. C., Moenke G. (2014). Reliable encoding of stimulus intensities within random sequences of intracellular Ca^2+^ spikes. *Science Signaling*.

[29] Aguado F., Espinosa-Parrilla J. F., Carmona M. A., Soriano E. (2002). Neuronal activity regulates correlated network properties of spontaneous calcium transients in astrocytes in situ. *The Journal of Neuroscience*.

[30] Pasti L., Volterra A., Pozzan T., Carmignoto G. (1997). Intracellular calcium oscillations in astrocytes: a highly plastic, bidirectional form of communication between neurons and astrocytes in situ. *The Journal of Neuroscience*.

[31] Zonta M., Angulo M. C., Gobbo S. (2003). Neuron-to-astrocyte signaling is central to the dynamic control of brain microcirculation. *Nature Neuroscience*.

[32] Bezzi P., Carmignoto G., Pasti L. (1998). Prostaglandins stimulate calcium-dependent glutamate release in astrocytes. *Nature*.

[33] Araque A., Li N., Doyle R. T., Haydon P. G. (2000). SNARE protein-dependent glutamate release from astrocytes. *The Journal of Neuroscience*.

[34] Pasti L., Zonta M., Vicini S., Carmignoto G. (2001). Cytosolic calcium oscillations in astrocytes may regulate exocytotic release of glutamate. *Journal of Neuroscience*.

[35] Panatier A., Theodosis D. T., Mothet J.-P. (2006). Glia-derived d-serine controls nmda receptor activity and synaptic memory. *Cell*.

[36] Queiroz G., Gebicke-Haerter P. J., Schobert A., Starke K., Von Kügelgen I. (1997). Release of ATP from cultured rat astrocytes elicited by glutamate receptor activation. *Neuroscience*.

[37] Cotrina M. L., Lin J. H.-C., Alves-Rodrigues A. (1998). Connexins regulate calcium signaling by controlling ATP release. *Proceedings of the National Academy of Sciences of the United States of America*.

[38] Guthrie P. B., Knappenberger J., Segal M., Bennett M. V. L., Charles A. C., Kater S. B. (1999). ATP released from astrocytes mediates glial calcium waves. *The Journal of Neuroscience*.

[39] Anderson C. M., Bergher J. P., Swanson R. A. (2004). Atp-induced atp release from astrocytes. *Journal of Neurochemistry*.

[40] Eddelston M., Mucke L. (1993). Molecular profile of reactive astrocytes—implications for their role in neurologic disease. *Neuroscience*.

[41] Li N., Sul J.-Y., Haydon P. G. (2003). A calcium-induced calcium inux factor, nitric oxide, modulates the refilling of calcium stores in astrocytes. *Journal of Neuroscience*.

[42] van den Pol A. N., Finkbeiner S. M., Cornell-Bell A. H. (1992). Calcium excitability and oscillations in suprachiasmatic nucleus neurons and glia in vitro. *The Journal of Neuroscience*.

[43] Charles A. C. (1994). Glia-neuron intercellular calcium signaling. *Developmental Neuroscience*.

[44] Manning T. J., Sontheimer H. (1997). Spontaneous intracellular calcium oscillations in cortical astrocytes from a patient with intractable childhood epilepsy (rasmussen's encephalitis). *Glia*.

[45] Tashiro A., Goldberg J., Yuste R. (2002). Calcium oscillations in neocortical astrocytes under epileptiform conditions. *Journal of Neurobiology*.

[46] Parri H. R., Crunelli V. (2003). The role of Ca^2+^ in the generation of spontaneous astrocytic Ca^2+^ oscillations. *Neuroscience*.

[47] Zur Nieden R., Deitmer J. W. (2006). The role of metabotropic glutamate receptors for the generation of calcium oscillations in rat hippocampal astrocytes in situ. *Cerebral Cortex*.

[48] Gimpl G., Kirchhoff F., Lang R. E., Kettenmann H. (1993). Identification of neuropeptide Y receptors in cultured astrocytes from neonatal rat brain. *Journal of Neuroscience Research*.

[49] Heinrich R., Schuster S. (1996). *The Regulation of Cellular Systems*.

[50] Dode L., Van Baelen K., Wuytack F., Dean W. L. (2001). Low temperature molecular adaptation of the skeletal muscle sarco(endo)plasmic reticulum Ca^2+^-atpase 1 (serca 1) in the wood frog (*Rana sylvatica*). *Journal of Biological Chemistry*.

[51] Rüdiger S., Shuai J. W., Huisinga W. (2007). Hybrid stochastic and deterministic simulations of calcium blips. *Biophysical Journal*.

[52] Ozisik M. (1993). *Heat Conduction*.

[53] Beck J., Cole K., Haji-Sheikh A., Litkouhi B. (1992). *Heat Conduction Using Green’s Function*.

[54] De Young G. W., Keizer J. (1992). A single-pool inositol 1,4,5-trisphosphate-receptor-based model for agonist-stimulated oscillations in Ca^2+^ concentration. *Proceedings of the National Academy of Sciences of the United States of America*.

[55] Bentele K., Falcke M. (2007). Quasi-steady approximation for ion channel currents. *Biophysical Journal*.

[56] Araque A., Carmignoto G., Haydon P. G. (2001). Dynamic signaling between astrocytes and neurons. *Annual Review of Physiology*.

[57] Pasti L., Pozzan T., Carmignoto G. (1995). Long-lasting changes of calcium oscillations in astrocytes: a new form of glutamate-mediated plasticity. *The Journal of Biological Chemistry*.

[58] Laskey A. D., Roth B. J., Simpson P. B., Russell J. T. (1998). Images of Ca^2+^ flux in astrocytes: evidence for spatially distinct sites of Ca^2+^ release and uptake. *Cell Calcium*.

[59] Falcke M., Li Y., Lechleiter J. D., Camacho P. (2003). Modeling the dependence of the period of intracellular Ca^2+^ waves on SERCA expression. *Biophysical Journal*.

[60] Meyer T., Holowka D., Stryer L. (1988). Highly cooperative opening of calcium channels by inositol 1,4,5-trisphosphate. *Science*.

[61] Dupont G., Goldbeter A., Goldbeter A. (1989). Theoretical insights into the origin of signal-induced calcium oscillations. *Cell to Cell Signalling*.

[62] Bertram R., Satin L. S., Pedersen M. G., Luciani D. S., Sherman A. (2007). Interaction of glycolysis and mitochondrial respiration in metabolic oscillations of pancreatic islets. *Biophysical Journal*.

[63] Falcke M. (2004). Reading the patterns in living cells—the physics of Ca^2+^ signaling. *Advances in Physics*.

[64] Mak D.-O. D., McBride S. M. J., Foskett J. K. (2003). Spontaneous channel activity of the inositol 1,4,5-trisphosphate (InsP3) receptor (InsP3R). Application of allosteric modeling to calcium and InsP3 regulation of the InsP3R single-channel gating. *Journal of General Physiology*.

[65] Cai L., Dalal C. K., Elowitz M. B. (2008). Frequency-modulated nuclear localization bursts coordinate gene regulation. *Nature*.

[66] McAnulty J. F., Ametani M. S., Southard J. H., Belzer F. O. (1996). Effect of hypothermia on intracellular Ca^2+^ in rabbit renal tubules suspended in UW-gluconate preservation solution. *Cryobiology*.

[67] Kim J.-S., Southard J. H. (1998). Alteration in cellular calcium and mitochondrial functions in rat liver during cold preservation. *Transplantation*.

[68] Haddad P., Cabrillac J.-C., Piche D., Musallam L., Huet P.-M. (1999). Changes in intracellular calcium induced by acute hypothermia in parenchymal, endothelial, and Kupffer cells of the rat liver. *Cryobiology*.

